# The Tumor-Suppressive Role of SAT2 in Pancreatic Cancer: Involvement in PI3K/Akt-MAPK Pathways and Immune Modulation

**DOI:** 10.3390/cimb47100872

**Published:** 2025-10-21

**Authors:** Ben Zhao, Lu Wang, Rui Fang, Xiaoxiao Luo, Lu Zhang

**Affiliations:** 1 Department of Oncology, Tongji Hospital, Tongji Medical College, Huazhong University of Science and Technology, Wuhan 430030, China; zhaoben0609@163.com (B.Z.); wanglu@hust.edu.cn (L.W.); xxluo2happy@hust.edu.cn (X.L.); 2 Bridge Institute of Experimental Tumor Therapy, West German Cancer Center, University Hospital Essen, University of Duisburg-Essen, 45147 Essen, Germany; rui.fang@dkfz-heidelberg.de; 3 Division of Solid Tumour Translational Oncology, German Cancer Consortium (DKTK), Partner Site Essen, a Partnership Between German Cancer Research Centre (DKFZ) and University Hospital Essen, 45147 Essen, Germany

**Keywords:** pancreatic cancer, spermidine/spermine N1-Acetyltransferase 2, prognosis, immune infiltration, invasion, migration

## Abstract

Spermidine/spermine N1-Acetyltransferase 2 (SAT2), belonging to the spermidine/spermine N1-Acetyltransferase family, has been increasingly recognized for its potential effects on tumor occurrence and development. Nonetheless, little is known about its biological function and clinical value for pancreatic cancer (PC). The present work focused on investigating its expression pattern, prognostic value, molecular mechanisms, and immune relevance in PC. SAT2 expression within PC samples and its prognostic significance were analyzed by retrieving the relevant data from the TCGA, CPTAC, and HPA databases. The biological function of SAT2 was investigated through GO and KEGG enrichment analyses. The association of SAT2 with immune cell infiltration in tumors was assessed by CIBERSORT. Additionally, in vitro experiments were performed to examine how SAT2 expression affected the PC cell proliferation, invasion, and migration abilities. An in vivo xenograft tumor model was employed for investigating how SAT2 expression affected the PC cell-derived tumor growth. The expression of SAT2 within the PC tissue exhibited a significant decrease in comparison with a non-carcinoma sample. Such observation was validated within PC cells. In addition, SAT2 expression showed a close relation to both tumor growth (T stage) and prognosis. SAT2 primarily participates in pathways, including the PI3K/Akt and MAPK pathways. Furthermore, we demonstrated a significant association between SAT2 expression and immune cell infiltration into tumors. The in vitro experiments confirmed that elevated SAT2 expression significantly suppressed PC cell viability, invasion, and migration through modulating the PI3K/Akt and MAPK pathways. The in vivo experimental results suggested the role of SAT2 overexpression in inhibiting xenograft tumor growth. Our investigation confirms the role of SAT2 in PC development through involvement in the PI3K/Akt and MAPK pathways. The correlation between SAT2 expression levels, immune infiltration, and checkpoint regulation provides valuable insights for immunotherapy strategies targeting PC.

## 1. Introduction

Pancreatic cancer (PC) stands as a fatal cancer, characterized by poor diagnostic accuracy and dismal patient prognosis. Additionally, the highly invasive nature of PC cells poses significant obstacles for achieving radical operation [1]. Despite the advancements in treatment modalities for PC, including chemotherapy or immunotherapy, its global 5-year survival rate remains at approximately 10% [2]. Understanding the mechanisms of PC development is important for the discovery of new therapeutic targets.

Spermidine/spermine N1-Acetyltransferase 2 (SAT2), also known as SAT2 or SAT-2, is a protein containing 170 amino acids and its molecular weight is approximately 20 kDa. It belongs to the spermidine/spermine N1-Acetyltransferase (SAT) family, alongside SAT1, and it was initially discovered due to its homology to SAT1 [3,4,5]. Although SAT1 is a key rate-limiting enzyme in the polyamine catabolic pathway, maintaining intracellular homeostasis and preventing polyamine toxicity [6], SAT2 exhibits markedly reduced acetylation activity toward polyamines [3,4]. Previous studies confirmed that SAT2 does not directly participate in polyamine metabolism and instead fulfills physiological functions distinct from SAT1 [5]. For example, SAT2 participates in the ubiquitin ligase complex regulating hypoxia-inducible factor 1α (HIF-1α), thus enhancing ubiquitination and decomposition [7,8]. SAT2 is probably implicated in tumor biology. The underexpression of SAT2 has been reported in colon cancer [9], raising the possibility of its tumor-suppressive role. However, its expression pattern, biological functions, and clinical significance in other malignancies remain poorly characterized, particularly in pancreatic cancer. This gap in knowledge prompted our investigation.

The present work investigated SAT2 expression in PC as well as the prognostic significance. Additionally, the relation of SAT2 with the immune response of PC was analyzed. Our hypothesis was that the upregulation of SAT2 may suppress PC cell migration and viability through suppressing the MAPK and PI3K/Akt pathways, thus impacting PD-L1 levels. These results shed additional light on the function of SAT2 in PC and its underlying mechanisms, indicating that the activation of SAT2 is a promising way to manage PC.

## 2. Materials and Methods

### 2.1. PC Sample Source

We collected transcriptome and clinical data from publicly available sources such as Human Protein Atlas (HPA, https://www.proteinatlas.org/, accessed on 24 April 2024) [10], The Cancer Genome Atlas (TCGA, https://portal.gdc.cancer.gov/, accessed on 11 April 2024 ) [11], UCSC Xena Browser (https://xenabrowser.net/, accessed on 17 April 2024) [12], and Clinical Proteomic Tumor Analysis Consortium (CPTAC, https://proteomic.datacommons.cancer.gov/, accessed on 26 April 2024) [13]. For pancreatic adenocarcinoma (PAAD) and other cancers, we retrieved RNA sequencing data with FPKM values, which were later transformed into TPM values prior to log_2_ transformation. Additionally, as for GSE16515, GSE15471, and GSE62165 datasets, their normalized matrix files were directly acquired based on Gene Expression Omnibus (GEO, https://www.ncbi.nlm.nih.gov/geo/, accessed on 20 April 2024 ) [14]. All analyses were conducted using R language (version 4.3.3). The clinical samples and patient information utilized in this study were obtained from publicly accessible databases, thereby exempting the need for review by the Ethics Committee.

### 2.2. Pathway and Gene Enrichment Analyses

The R “corrr” package was utilized for identifying genes that were co-expressed with SAT2, considering a correlation coefficient > 0.3 or <−0.3 and *p* < 0.05. The R “clusterProfiler” package was utilized to perform Gene Ontology (GO) as well as Kyoto Encyclopedia of Genes and Genomes (KEGG) enrichment. The R package “ggplot2” was applied in visualizing the most statistically significant GO terms together with KEGG pathways. Our enrichment analysis encompassed Biological Process (BP) and Molecular Function (MF), along with Cellular Component (CC) terms, using *p* < 0.05 as the significance level in GO and KEGG enrichments.

### 2.3. Analysis of Infiltration of Immune Cells

We used “CIBERSORT” software for evaluating 22 distinct immune cell infiltration statuses within PC [15]. Correlations of genes with immune infiltration scores within different tumors were determined using “corr.test” function from R package “psych”, identifying significant associations with immune infiltration scores.

### 2.4. Cell Culture and Transfection

Human healthy pancreatic ductal epithelial HPDE6-C7 cells were obtained from Xiamen Immocell Biotechnology Co., Ltd. (Xiamen, China) in June 2023. Additionally, PC cell lines (AsPC-1, Capan-1, SW1990, BxPC-3, and PANC-1) were obtained from the American Type Culture Collection (Manassas, VA, USA) in April 2023. The authenticity of all cell lines was confirmed through Short Tandem Repeat analysis, while regular PCR assays were conducted every 3 months to ensure the absence of mycoplasma contamination.

We kept cell cultures at 37 °C with a CO_2_ concentration of 5% in either RPMI-1640 or DMEM that contained 10% fetal bovine serum (Cat. No. A5256701FBS, Gibco, Grand Island, NY, USA). The SAT2 overexpression plasmid (pcDNA3-SAT2) was constructed by cloning the full-length coding sequence (CDS) of human SAT2 (NM_001320845.1) into the pcDNA3-mRFP vector using standard restriction–ligation methods (EcoRI/XhoI), with empty pcDNA3-mRFP vector being the negative control (pcDNA3-NC). Lipofectamine^TM^ 2000 Reagent (Cat. No. 11668019, Thermo Fisher Scientific, Waltham, MA, USA) was used to transfect pcDNA3-SAT2 and pcDNA3-NC in PANC-1 and BxPC-3 cells following specific protocols. At 48 h after transfection, SAT2 expression within cells was measured through qRT-PCR together with Western blotting techniques to validate the efficiency of overexpression.

### 2.5. qRT-PCR Assay

Gene levels were detected through qRT-PCR. Specifically, we used TRIzol reagent (Cat. No. DP424, TianGen, Beijing, China) to isolate total cellular RNA and later adopted MightyScript First Strand cDNA Synthesis Master Mix (Cat. No. B639251, Sangon Biotech, Shanghai, China) to convert it to cDNA. The cDNA was utilized to be the template in PCR amplification, and SYBR Green Real-Time PCR Master Mix (Cat. No. A46109, Thermo Fisher Scientific, Waltham, MA, USA) was employed to prepare the PCR system. Our reaction protocol consisted of 10 min pre-denaturation at 95 °C, 15 s denaturation at 95 °C, 30 s annealing at 60 °C, and 30 s extension at 72 °C, for a total of 40 cycles. Primer sequences utilized are shown below. SAT2 Forward: 5′-ATCCTGAGGCTGATTCGGGA-3′, Reverse: 5′-TC CTCCAGAT AAATGGTGCGT-3′; GAPDH Forward: 5′-AGCCACATCGCTCAGA CAC-3′, Reverse: 5′-GCCCAATACGACCAAATCC-3′. SAT2 mRNA expression in cells was quantified by the 2^−ΔΔCt^ approach, and GAPDH was the control.

### 2.6. CCK-8 Assay

Cell proliferation was assessed using Cell Counting Kit-8 (CCK-8) (Cat. No. E-CK-A362, Elabscience Biotechnology, Wuhan, China). To be specific, cells (2 × 10^3^/well) were inoculated into a 96-well plate, followed by incubation for 12, 24, 48, and 72 h. Following each time interval, 10 μL CCK-8 solution was added and incubated for 2 h. Subsequently, optical density (OD) values was measured at 450 nm.

### 2.7. EdU (5-Ethynyl-2′-Deoxyuridine) Assay

The EdU assay was carried out following the instructions provided with the EdU Kit (Cat. No. C10338, Thermo Fisher Scientific, Waltham, MA, USA). Cells (5 × 10^4^/well) were inoculated into a 12-well plate and cultured for 24 h to achieve complete adhesion. The cells later experienced 2 h of incubation within the medium that contained EdU solution (50 μM). After 20 min of cell fixation with 4% paraformaldehyde at ambient temperature, DAPI solution (1 µg/mL) was used to stain cells for a 10 min duration. EdU-positive cell proportion was then determined by analyzing five random fields under a microscope.

### 2.8. Wound Healing Assay

We cultured the logarithmically growing cells (5 × 10^4^/well) into the six-well plate. After a confluent monolayer was formed, vertical scratches were created using a 200 μL pipette tip. Later, PBS was added to rinse suspended cells for removal, followed by incubation in serum-free medium (SFM) at 37 °C and 5% CO_2_ for 24 h. Microscopic images of the scratched area were captured at both 0 and 24 h time points, followed by calculation of scratch area using ImageJ 2 software (NIH, Bethesda, MD, USA) to determine wound healing rate.

### 2.9. Transwell Assay

For invasion assays, Matrigel matrix (100 μL, Corning Incorporated, Corning, NY, USA) was added to upper Transwell^®^ insert chambers (8 μm, Corning Incorporated, Corning, NY, USA). After dilution with SFM to 2 × 10^5^ cells/mL, cell suspension (200 μL) was introduced into the upper chamber, whereas medium (600 μL) containing 20% FBS was added to the bottom chamber. At 24 h post-incubation at 37 °C, upper membrane surface was wiped with a cotton tip, followed by application of Giemsa stain onto the lower surface. After staining, cell number was counted from five random microscopic fields, and the mean value was utilized for statistical analysis.

### 2.10. Western Blotting

The harvested cells underwent lysis using RIPA buffer (Solarbio, Beijing, China). Protein content in the cell lysates was analyzed with BCA Kit (Beyotime Biotechnology, Shanghai, China). Following quantification, protein separation was conducted on SDS-PAGE gels, followed by transfer onto PVDF membranes. After blocking using 5% defatted milk, membranes experienced primary antibody incubation overnight, including anti-SAT2 (Cat. No. NBP1-80722, Novus Biologicals, Centennial, CO, USA), anti-Cyclin D1 (Cat. No. ab16663, Abcam, Cambridge, UK), anti-AKT (Cat. No. #9272), anti-Phospho-Akt (Ser473) (Cat. No. #4060), anti-Bad (Cat. No. #9292), anti-Vimentin (Cat. No. #5741), anti-E-cadherin (Cat. No. #3195), anti-p38 MAPK (Cat. No. #9212), anti-Erk1/2 (Cat. No. #4696), anti-Phospho-p38 MAPK (Thr180/Tyr182) (Cat. No. #9211), anti-PD-L1 (Cat. No. #13684), anti-GAPDH (Cat. No. #2118), anti-Phospho-STAT3 (Tyr705) (Cat. No. #9145), anti-STAT3 (Cat. No. #9139), and anti-Phospho-Erk1/2 (Thr202/Tyr204) (Cat. No. #4370) antibodies (CST, Danvers, MA, USA) at 4 °C. Following a rinse using TBST solution, the membranes were then exposed to corresponding HRP-conjugated secondary antibodies for 1 h at ambient temperature. Finally, the ECL Detection Kit (Cat. No. PE0010, Solarbio, Beijing, China) was utilized for visualizing protein bands, followed by quantification using ImageJ software (NIH, Bethesda, MD, USA).

### 2.11. Co-Culture System

Human peripheral blood mononuclear cells (PBMCs, Cat. No.CP-H182) were purchased from Wuhan Pricella Biotechnology Co., Ltd. (Wuhan, China). PBMCs were cultured in vitro using complete medium and pre-stimulated with human CD3/CD28 antibodies for activation. Subsequently, the PBMC concentration was adjusted to 4 × 10^6^ cells/mL. PANC-1 and BxPC-3 cells in the logarithmic growth phase were harvested, and their concentration was adjusted to 2 × 10^5^ cells/mL. A volume of 1 mL of this cell suspension was seeded into each well of a 6-well plate. Following complete cell adhesion, 0.5 mL of the PBMC suspension was added at an effector-to-target (E:T) cell ratio of 10:1. The co-culture system was incubated at 37 °C in a humidified atmosphere containing 5% CO_2_ for 24 h.

### 2.12. Cytotoxicity Detection

Following completion of the co-culture, the supernatant was collected and processed in accordance with the manufacturer’s instructions for the LDH Cytotoxicity Assay Kit (Cat. No. C0018S, Beyotime, Shanghai, China). A volume of 100 μL of supernatant was transferred from each sample group into a 96-well plate. An equal volume (100 μL) of LDH assay working solution was added to each well, followed by thorough mixing. The plate was then incubated at room temperature in the dark for 30 min. Subsequently, 20 μL of stop solution was added to each well to terminate the reaction, followed by gentle mixing. Absorbance was measured at 450 nm using an ELISA microplate reader (Thermo Fisher Scientific, Waltham, MA, USA). The cytotoxicity (%) was calculated using the following formula: [(A_sample − A_blank control)/(A_maximum enzyme activity control − A_blank control)] × 100%.

### 2.13. Enzyme-Linked Immunosorbnent Assay (ELISA)

After completion of the co-culture, the supernatant was collected and we proceeded with analysis using the Human IL-10 ELISA Kit (Cat. No. E-EL-H6154) and TGF-β1 ELISA Kit (Cat. No. E-EL-0162), both from Elabscience (Wuhan, China), following the manufacturer’s instructions. For TGF-β1 detection, activation of the latent form was required prior to assay. Absorbance at 450 nm was measured using an ELISA microplate reader, and the concentrations of IL-10 and TGF-β1 in the supernatant were determined based on the respective standard curves.

### 2.14. Xenograft Tumor Model

The SAT2-overexpressing lentivirus (Lv-SAT2, titer: 3.57 × 10^8^ TU/mL) and the corresponding negative control lentivirus (Lv-NC, titer: 7.26 × 10^8^ TU/mL) were provided by Hanbio Biotechnology (Shanghai, China) Co., Ltd. Briefly, the CDS of the SAT2 gene (NM_001320845.1) was targeted for PCR primer design to amplify the SAT2 gene fragment. The primers used for amplification were as follows: SAT2-F: 5′-GCG ACCGGTATGCCCGGACAGAGGATCGC-3′; SAT2-R: 5′-GCGGAATTCACA TGTAATTCTTATTTATTTTCACC-3′. Both the pHBLV-CMV-MCS-EF1-Puro plasmid and the PCR-amplified product were subjected to double digestion with the restriction enzymes AgeI and EcoRI. The digested products were separated by agarose gel electrophoresis, followed by gel extraction to recover the linearized vector and the SAT2 gene insert. The purified SAT2 fragment was ligated into the linearized vector using T4 DNA ligase to generate the recombinant plasmid. This construct was then transformed into competent *Escherichia coli* DH5α cells. Transformed bacteria were plated onto culture dishes containing ampicillin, and positive colonies were selected and verified by colony PCR and DNA sequencing. The correctly sequenced recombinant plasmid, designated pHBLV-CMV-SAT2-EF1-Puro (10 μg), was co-transfected with the lentiviral packaging plasmids psPAX2 (7.5 μg) and pMD2.G (2.5 μg) into HEK293T cells. After 48 h of cell infection, the cell culture supernatant containing lentiviral particles was collected. The viral stock solution was filtered through a 0.45 μm membrane to remove cellular debris, followed by concentration and purification steps, and the viral titer was subsequently determined.

The lentiviruses for Lv-SAT2 and Lv-NC were used to infect PANC-1 cells (MOI = 100). After 72 h, 2 μg/mL puromycin was added to select stably transfected cells. Ten BALB/c nude mice (4–6 weeks old, weighing 18–22 g) were purchased from Wuhan Bestcell Model Biotechnology Co., Ltd. (Wuhan, China). After one week of acclimatization, the mice were randomly assigned into two groups using a random number table method, ensuring unbiased allocation between experimental and control conditions. To maintain objectivity in intervention administration and observation, the operator responsible for cell suspension preparation and subcutaneous injection was blinded to the identity of the transfectants prior to injection. PANC-1 stable transfectants overexpressing SAT2 (Lv-SAT2) and the negative control (Lv-NC) were resuspended in serum-free DMEM medium and adjusted to a density of 2 × 10^7^ cells/mL. A 100 μL cell suspension was subcutaneously injected into the axillary area of the right forelimb of each mouse to establish a PC xenograft tumor model. The mice were monitored for 21 days, and tumor length and width were measured on days 5, 9, 13, 17, and 21. Tumor volume was calculated using the following formula: Volume = Length × Width^2^ × 0.52. At the endpoint, the mice were euthanized by CO_2_ asphyxiation, and tumor tissues were promptly dissected, weighed, and fixed in 4% paraformaldehyde for 48 h for subsequent paraffin embedding and histopathological analysis. The animal experiments of this study were approved by the Laboratory Animal Welfare and Ethics Review Committee of Hubei BenNT Experimental Center (Ethics Approval No. BNB-2025-006).

### 2.15. Hematoxylin and Eosin (HE) Staining

After fixation, tumor tissues underwent sectioning into 4 μm thick paraffin slices. Following the manufacturer’s protocol for the H&E Staining Kit (Cat. No. C0105S, Beyotime, Shanghai, China), morphological features of the tumor tissues were observed and imaged under a microscope.

### 2.16. Immunohistochemistry (IHC)

After antigen retrieval and blocking, tumor sections received overnight primary antibody incubation at 4 °C, including anti-SAT2 (1:200, Cat. No. 16246-1-AP, Proteintech, Rosemont, IL, USA), anti-PD-L1 (1:200, Cat. No. #13684, CST, Danvers, MA, USA), and anti-Ki-67 (1:100, Cat. No. NBP2-22112, Novus Biologicals, Centennial, CO, USA). The next day, the corresponding secondary antibodies were applied to the sections for 30 min at 37 °C, followed by DAB chromogenic development and hematoxylin counterstaining. Stained sections were monitored using a microscope, and an integrated optical density (IOD) of positive staining was quantified using Image J2 software (NIH, Bethesda, MD, USA). SAT2 immunoreactivity was predominantly localized in the cytoplasm and nucleus, while Ki-67 staining was nuclear, with positive signals appearing brownish-yellow.

### 2.17. TUNEL Assay

Paraffin sections were treated with proteinase K for 20 min. Following the TUNEL Staining Kit (Cat. No.C1088, Beyotime, Shanghai, China) protocol, TUNEL reaction mixture was added and incubated for 1 h in the dark at 37 °C. After 5 min of DAPI nuclear counterstaining, images were captured with a fluorescence microscope. The apoptotic index (TUNEL-positive to total cell ratio) was determined using ImageJ software for analyzing how SAT2 affected tumor cell apoptosis.

### 2.18. Statistical Analysis

Statistical analysis was completed using SPSS 22.0 software. The normality of the distribution for all continuous data was assessed using the Shapiro–Wilk test. Data that passed the normality test and homogeneity of variances (assessed by Levene’s test) are presented as mean ± standard deviation and were analyzed using parametric tests. Specifically, the Student’s *t*-test was used for comparisons between two groups, and one-way ANOVA was used for comparisons among multiple groups. The Wilcoxon rank-sum test was applied for non-parametric data comparisons, such as gene expression levels from databases. Correlations were assessed using Spearman’s correlation coefficient. Kaplan–Meier curve analysis was conducted, while log-rank test was adopted for evaluating differences. To identify significant factors that independently influenced prognosis, univariate as well as multivariate Cox regression was carried out with R “survival” package. *p* < 0.05 stood for statistical significance.

## 3. Results

### 3.1. SAT2 Expression Is Low and Related to PC Tumor Size

**Figure 1:**
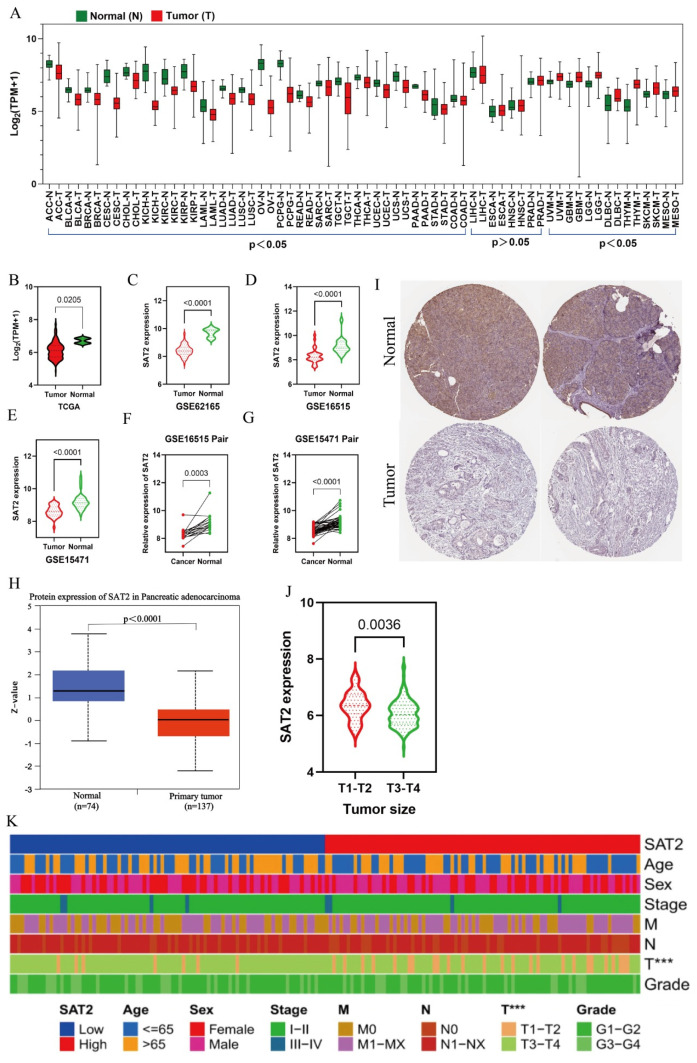
SAT2 expression is low in PC. (**A**) SAT2 expression within diverse samples in TCGA and GTEx datasets; (**B**) SAT2 mRNA expression inside TCGA-derived PC tissues; (**C**–**E**) SAT2 expression within GSE62165, GSE16515, and GSE15471 cohorts; (**F**,**G**) expression profiles of pairs of PC tissues and normal tissues in GSE16515 and GSE15471 cohorts; (**H**,**I**) protein level of SAT2 analyzed using the CPTAC and HPA databases; (**J**) SAT2 expression in different T stages (tumor sizes) in PAAD using the TCGA database; (**K**) association between SAT2 expression and clinical parameters. *** *p* < 0.01.

Based on data obtained from the TCGA and GTEx databases, we conducted a comparative analysis of SAT2 gene expression levels in various human cancers and normal tissues. Table 1 presents the names of the tumors and the corresponding sample numbers. Our findings indicate that SAT2 mRNA expression markedly declined in multiple cancer tissues, including PC tissue, when compared to the expression observed in normal tissues (Figure 1A,B). The validation using the GSE62165, GSE16515, and GSE15471 datasets from GEO data further confirmed a significant decrease in SAT2 expression levels in PC (Figure 1C–E). The analysis of the paired samples from the GSE16515 (16 tumor samples and normal samples) and GSE15471 (39 tumor samples and normal samples) datasets further confirmed the low expression of SAT2 in PC tissues (Figure 1F,G). Additionally, the results from the CPTAC and HPA datasets demonstrated decreased protein expression levels of SAT2 in PC compared to the normal samples (Figure 1H,I), indicating the consistency of SAT2 mRNA and protein expression across multiple databases. According to the average expression level of SAT2, we classified patients with PC from the TCGA database as being in the SAT2 low-expression or SAT2 high-expression group (Table 2). The association between the SAT2 expression and the clinical parameters was investigated. The findings indicate a significantly different SAT2 expression between the patients classified as T1-T2 and those classified as T3–T4 (Figure 1J,K). These results collectively indicate the aberrant expression of SAT2 in PC.

### 3.2. Low Expression of SAT2 Predicts Poor PC Prognostic Outcome

**Figure 2:**
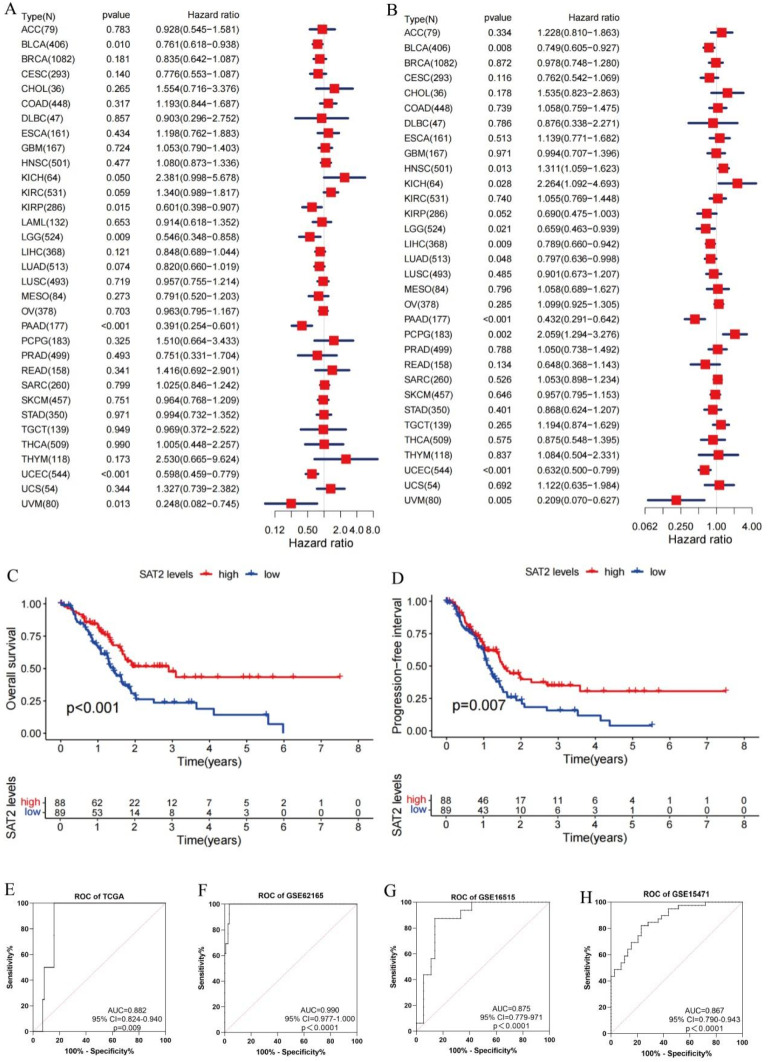
Prognostic and diagnostic value of SAT2 in PC. (**A**,**B**) Forest plot showing the relation of SAT2 expression with OS in 33 tumors and PFS in 32 tumors. The term “N” refers to the total number of cases across different tumors; (**C**,**D**) Kaplan–Meier analysis for the relation of SAT2 expression with OS and PFS; (**E**–**H**) ROC curve for assessing whether SAT2 expression was sensitive and specific as a diagnostic biomarker for PC in the TCGA, GSE62165, GSE16515, and GSE15471 databases.

Based on the TCGA dataset, we conducted an investigation into the correlation between SAT2 expression and the patients’ prognoses. We conducted an analysis of the patient outcomes, specifically focusing on overall survival (OS) as well as progression-free survival (PFS). Utilizing the Cox regression, the SAT2 level was significantly correlated with OS in six specific cancer types: BLCA, KIRP, LGG, PAAD, UVM and UCEC (Figure 2A). Furthermore, this study investigated the potential relation of the SAT2 level with PFS in patients with PC. Our results indicated that SAT2 expression had an impact on PFS in ten different cancers: BLCA, HNSC, KICH, LGG, LIHC, LUAD, PCPG, PAAD, UVM, and UCEC (Figure 2B). According to the Kaplan–Meier survival curves, a low SAT2 expression was strongly linked to unfavorable outcomes in both OS and PFS for the PAAD patients (Figure 2C,D). Additionally, to assess the value of SAT2 expression in diagnosis, we plotted an ROC curve for further examination. The AUC of the SAT2 level calculated from the TCGA sample was 0.882 (95%CI = 0.824~0.940), and similar findings were obtained from the GEO dataset, suggesting that the above results have high diagnostic application potential (Figure 2E–H).

### 3.3. SAT2 Expression Has Independent Prognostic Value in PC

**Figure 3:**
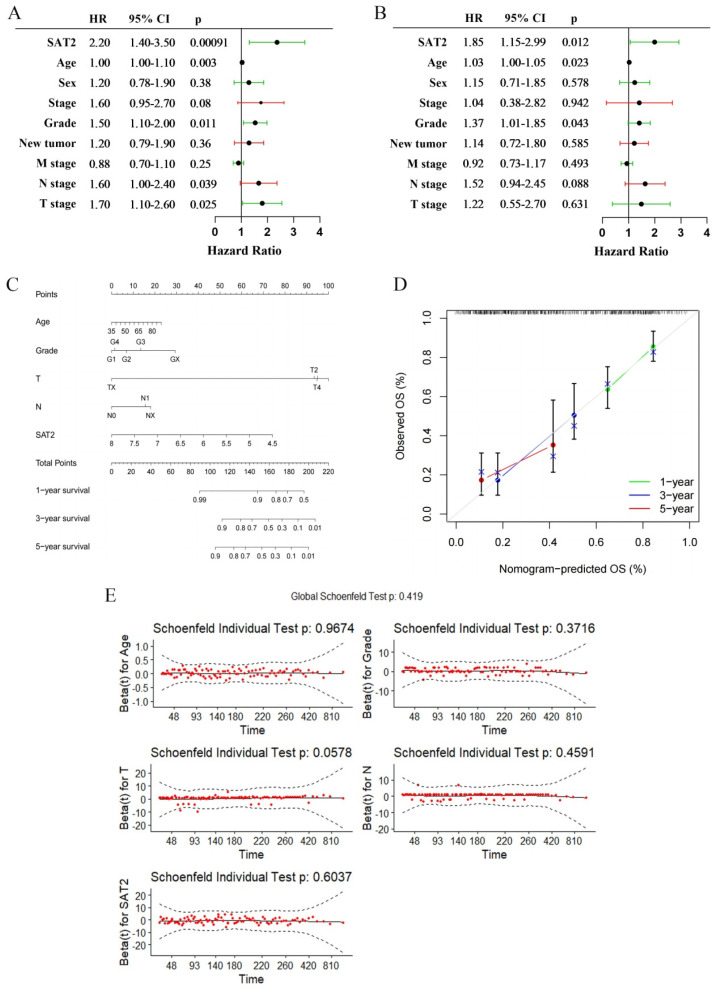
Independent prognostic analysis, nomogram establishment, and CoxPH model assumptions in PC. (**A**) Univariate and (**B**) multivariate Cox regression conducted to analyze SAT2 and clinical features; (**C**) the nomogram consists of age, grade, N stage (nodal metastasis status), T stage (tumor size), and SAT2 expression; (**D**) the calibration curve for evaluating model accuracy; (**E**) the Schoenfeld residuals test to examine the CoxPH model assumptions with age, grade, T stage (tumor size), N stage (nodal metastasis status), and SAT2 level.

Through univariate Cox regression analysis, it was found that a low expression of SAT2 is strongly related to a dismal OS (HR = 2.20, 95% CI = 1.40–3.50, *p* < 0.001) (Figure 3A). Furthermore, the multivariable regression analysis confirmed that SAT2 can independently predict PC prognosis (HR = 1.85, 95% CI = 1.15–2.99, *p* = 0.012) (Figure 3B). A nomogram based on age, grade, T stage (tumor size), N stage (nodal metastasis status), and SAT2 expression levels was developed to predict the survival rate at both one and two years in PC patients (Figure 3C), which accurately predicted the clinical outcomes at these time points, according to the calibration curves (Figure 3D). Additionally, a violation of proportional hazards could not be observed according to the test of the Schoenfeld residuals (*p* = 0.419, Figure 3E). Therefore, SAT2 is an important marker that predicts overall survival in PC patients.

### 3.4. SAT2 Is Related to PI3K/Akt and MAPK Pathways Through GSEA Analysis

**Figure 4:**
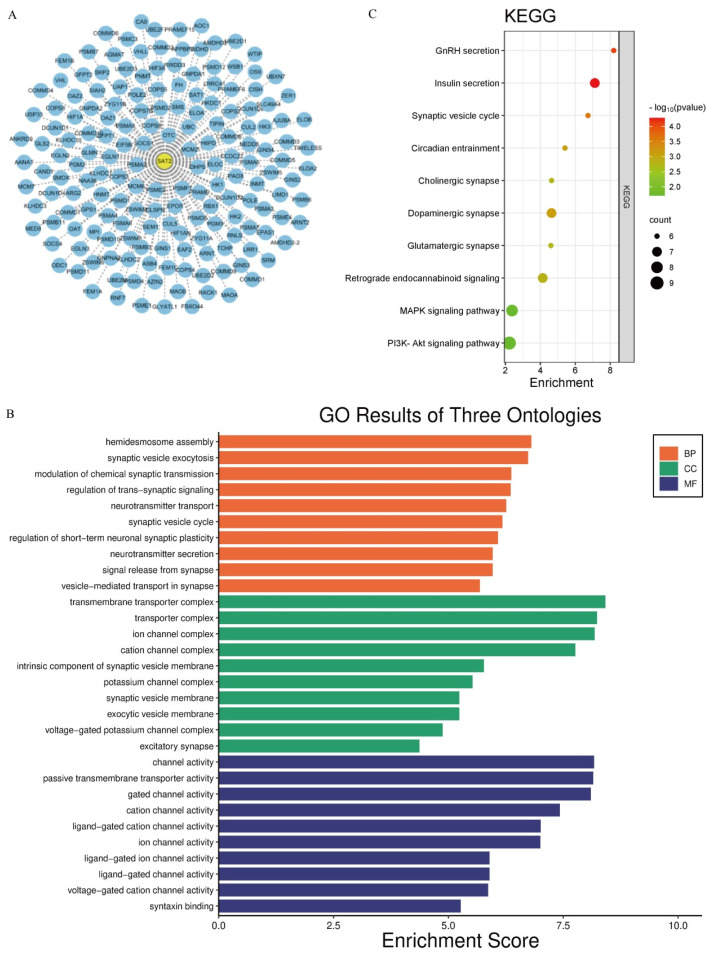
Co-expression network construction and enrichment analysis for SAT2. (**A**) The Cytoscape 3.10.3 platform was employed to construct an exquisite co-expression network encompassing 266 SAT2 co-expressed genes; (**B**) GO annotation for SAT2 according to SAT2 co-expressed genes; (**C**) KEGG enrichment for SAT2 according to SAT2 co-expressed genes.

For exploring SAT2’s biological effects on PC, GO enrichment as well as KEGG pathway analysis was implemented. Through Pearson correlation analysis (|*r*| > 0.3, *p* < 0.05), we identified 266 genes co-expressed with SAT2 across the PC datasets (Figure 4A). These genes were subsequently subjected to functional enrichment analysis. Based on the GO annotation, the SAT2-associated genes were closely associated with biological processes related to transmembrane transport, particularly in ion channels and small molecule transport systems (Figure 4B). Therefore, SAT2 is probably associated with cellular metabolic regulation and intracellular signaling dynamics. The KEGG pathway analysis further indicated that SAT2 is potentially implicated in multiple oncogenic pathways, including the PI3K/Akt and MAPK signaling pathways, as well as small molecule secretion pathways (Figure 4C). The PI3K/Akt and MAPK pathways are known to regulate various critical cellular processes, including cell proliferation, migration, apoptosis, and immune modulation. Given their pivotal roles in tumor progression and immune regulation, these findings suggest that SAT2 may influence the tumor microenvironment by modulating immune cell function. To further investigate this hypothesis, we next explored the relation of SAT2 expression to immune cell infiltration within pancreatic cancer.

### 3.5. SAT2 Level Is Related to Immune Cell Infiltration Within PC Samples

**Figure 5:**
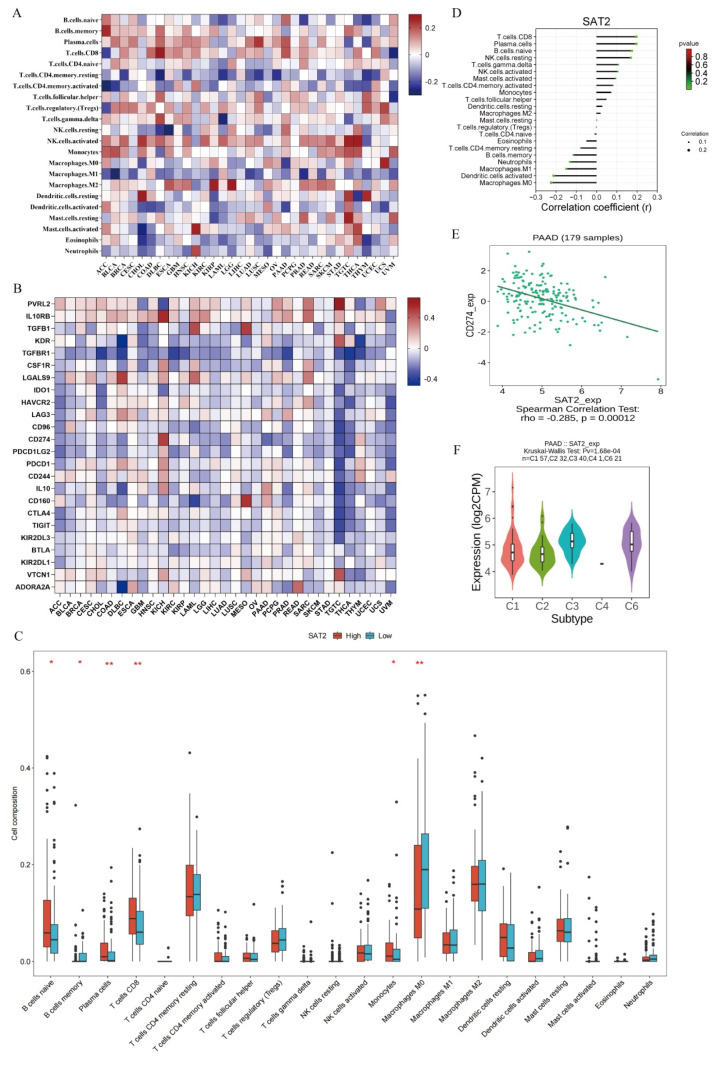
SAT2 expression is related to immune checkpoints and immune cell infiltration inside PC. (**A**) Correlations of SAT2 expression with immune cells in 33 cancer types; (**B**) associations of SAT2 expression with immune checkpoints; (**C**) immune cell infiltration levels in high- and low-SAT2 groups; (**D**) associations between SAT2 expression and immune cells; (**E**) relationship between SAT2 and PD-L1 expression; (**F**) associations of SAT2 expression with PC immune subtypes. * *p* < 0.05, ** *p* < 0.01.

To further investigate SAT2’s effect on the tumor immune microenvironment, its association with immune cell infiltration into PC was analyzed using the CIBERSORT algorithm. A total of 22 immune cell types and immune checkpoints were evaluated for their infiltration levels in relation to SAT2 expression (Figure 5A,B). We first compared the immune cell composition in SAT2 high- versus low-expression groups. Notably, M0 macrophages had a markedly increased abundance in the high-expression group, whereas plasma, CD8^+^ T, naive B, and memory B cells had significantly reduced abundances (Figure 5C). The correlation analysis further confirmed these findings, revealing a positive relationship between SAT2 expression and CD8^+^ T/naive B/plasma cells, whereas an inverse correlation was observed for M0 macrophages and activated dendritic cells (Figure 5D). Therefore, SAT2 is important for modulating immune cell infiltration within PC. Furthermore, we assessed the relation of SAT2 expression to immune checkpoint molecules to explore its potential involvement in immune evasion mechanisms. Interestingly, SAT2 expression was negatively related to PD-L1 (CD274) levels (*r* = −0.285, *p* = 0.00012) (Figure 5E), revealing that SAT2 may influence the immunosuppressive landscape of PC (Figure 5B,E).

To examine the impact of SAT2 on diverse PC immune subtypes, we utilized the TISIDB database, which classifies tumors into six immune subtypes: C1 (wound healing), C2 (IFN-γ dominant), C3 (inflammatory), C4 (lymphocyte-depleted), C5 (immunologically quiet), and finally, C6 (TGF-β dominant) [16]. The SAT2 expression varied significantly across these subtypes (*p* < 0.01), with the highest levels observed in the C4 (lymphocyte-depleted) and C6 (TGF-β dominant) subtypes, whereas the lowest expression was found in the C2 (IFN-γ dominant) as well as the C3 (inflammatory) subtypes (Figure 5F). This suggests that SAT2 may be preferentially expressed in immunosuppressive tumor environments. Collectively, these findings indicate that SAT2 is tightly related to immune cell infiltration, immune subtype classification, and immune checkpoint regulation in PC. Given its strong correlations with key immune components, SAT2 may have an important effect on shaping the tumor immune microenvironment, and the feasibility of using SAT2 as a therapeutic target should be further investigated.

### 3.6. Overexpression of SAT2 Inhibits PC Cell Growth, Invasion, and Migration

**Figure 6:**
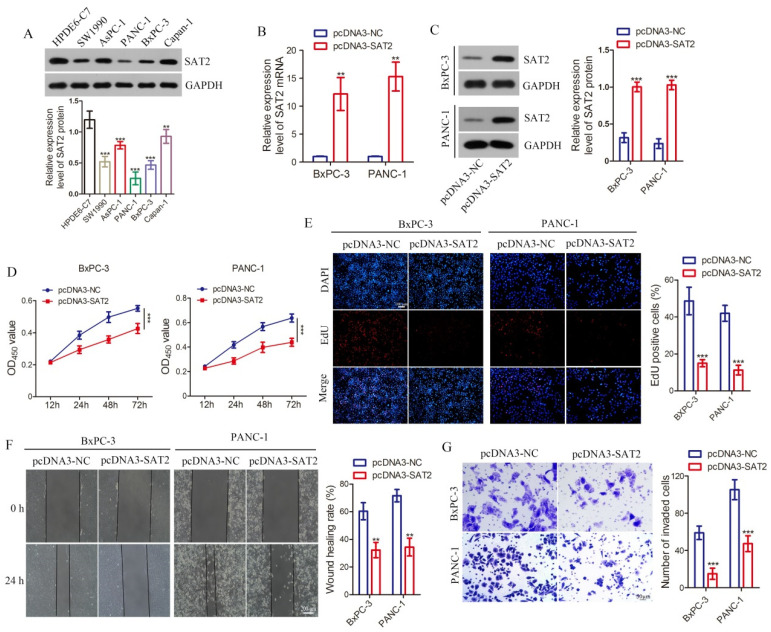
SAT2 high expression inhibits PC cell proliferation, migration, and invasion. (**A**) SAT2 protein levels within human healthy pancreatic ductal epithelial cells (HPDE6-C7) and PC cells (AsPC-1, Capan-1, SW1990, BxPC-3, and PANC-1); (**B**,**C**) SAT2 mRNA and protein levels within pcDNA3-SAT2-transfected BxPC-3 and PANC-1 cells; (**D**,**E**) cell viability in transfected BxPC-3 and PANC-1 cells determined through CCK8 assay and EdU assay (bar = 100 μm); (**F**,**G**) cell migration and invasion abilities of transfected BxPC-3 and PANC-1 were detected by wound healing (bar = 200 μm) and Transwell assays (bar = 50 μm). ** *p* < 0.01, *** *p* < 0.001 vs. HPDE6-C7 cells or pcDNA3-NC group.

To investigate SAT2 involvement in PC and elucidate its underlying molecular mechanism, we conducted in vitro cellular experiments. According to the results obtained from the western blot analysis, it was observed that the cancer cells exhibited lower levels of SAT2 protein compared to the normal cells in five different pancreatic cell types. Notably, PANC-1 and BxPC-3, two PC cell lines, displayed a particularly reduced expression of SAT2 (Figure 6A). Consequently, these two cell lines were selected for further experimentation. Subsequently, we transfected PANC-1 and BxPC-3 cells using a SAT2 overexpression plasmid, thus markedly increasing the SAT2 mRNA and protein levels (Figure 6B,C). As demonstrated by the results of the CCK-8 assays, SAT2 up-regulation markedly attenuated the PC cell proliferative activity time-dependently (Figure 6D). Additionally, our findings provide evidence suggesting that enhancing SAT2 overexpression leads to a notable reduction in cell proliferation, according to the EDU assay (Figure 6E). These discoveries support the notion that SAT2 has an important effect on regulating PC cell proliferation. Next, we evaluated the invasion and migration capacity of PC cells through the scratch and Transwell assays to investigate the function of SAT2. According to the results of the scratch experiments, SAT2 overexpression markedly decreased PC cell migration ability (Figure 6F). Based on the Transwell assay, PC cell invasion markedly decreased after SAT2 overexpression (Figure 6G). These findings imply that SAT2 potentially functions as a tumor suppressor gene in PC.

### 3.7. Overexpression of SAT2 Inhibits MAPK and PI3K/AKT Pathway Activation and PD-L1 Expression in PC Cells

**Figure 7:**
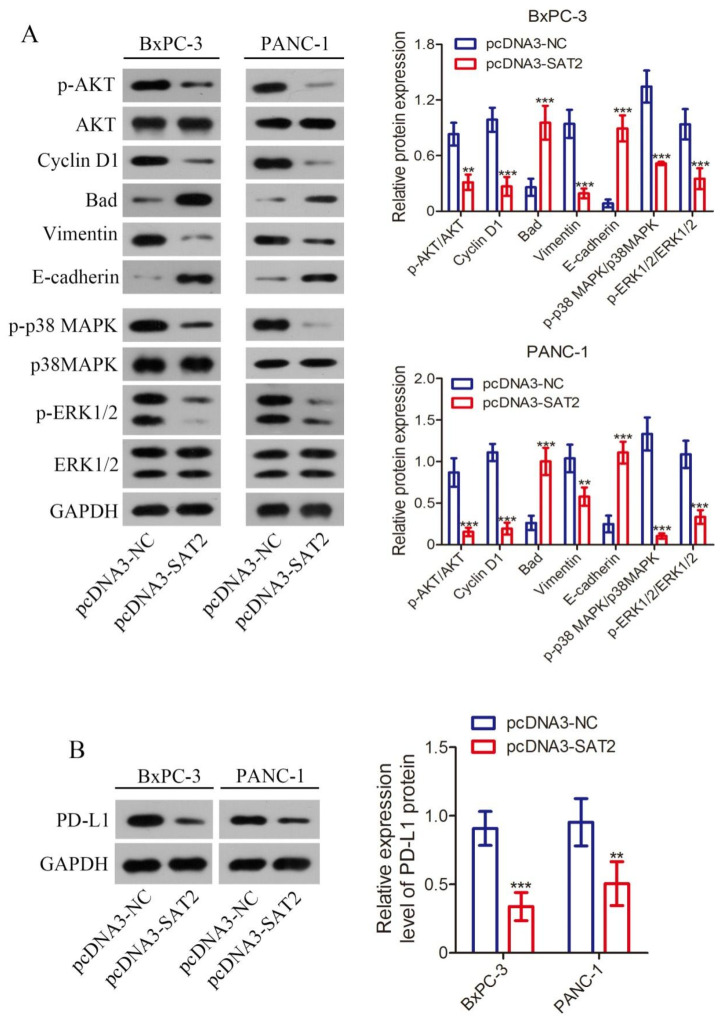
SAT2 high expression suppressed PI3K/Akt and MAPK pathway activation and PD-L1 expression. (**A**) MAPK and PI3K/Akt pathway related protein levels in transfected PANC-1 and BxPC-3 cells; (**B**) protein levels of PD-L1 in transfected PANC-1 and BxPC-3 cells. ** *p* < 0.01, *** *p* < 0.001 vs. pcDNA3-NC group.

**Figure 8:**
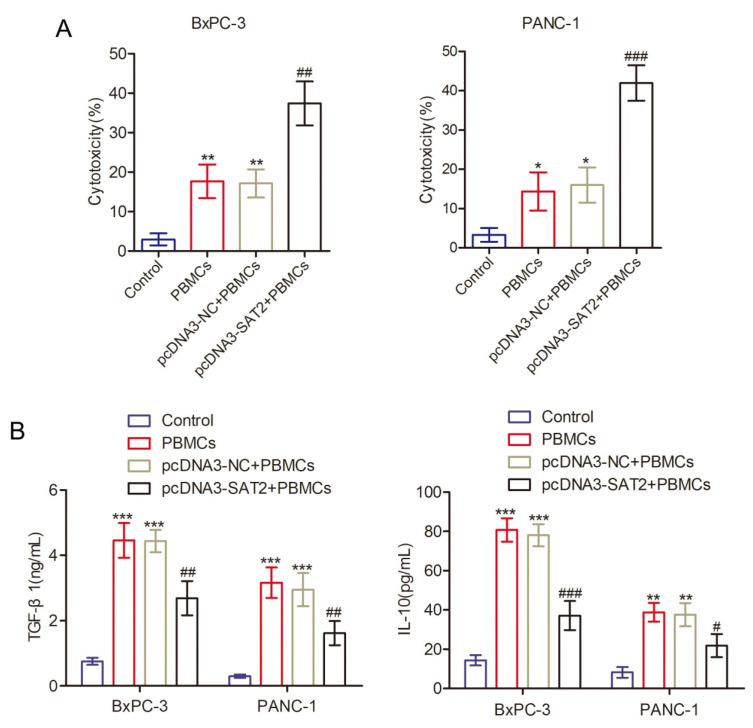
SAT2 high expression enhanced the susceptibility of PC cells to cytotoxicity of PBMCs. (**A**) The cytotoxicity of PBMCs against PC cells was indicated by the level of LDH release; (**B**) ELISA was used to detect the levels of immunosuppressive factors TGF-β1 and IL-10 in the cell supernatant. * *p* < 0.05, ** *p* < 0.01, *** *p* < 0.001 compared with the control group; ^# ^*p* < 0.05, ^##^ *p* < 0.01, ^###^ *p* < 0.001 compared with the pcDNA3-SAT2 + PBMCs group.

Our curiosity was sparked by the underlying mechanisms governing SAT2-mediated regulation of proliferation, migration, and invasion in PC cells. Based on our previous bioinformatics analysis, we formulated a hypothesis that the MAPK and PI3K/Akt pathways are involved. Consequently, in this study, we conducted additional validation experiments. As shown in Figure 7A, the overexpression of SAT2 within BxPC-3 and PANC-1 cells resulted in a significant reduction in key protein phosphorylation levels within the PI3K/Akt signaling pathway, such as Akt. Furthermore, the protein phosphorylation levels within the MAPK signaling pathway decreased, including p38MAPK and ERK. Additionally, downstream proteins within both pathways, such as Cyclin D1 and Vimentin, exhibited decreased expression levels while Bad and E-cadherin were upregulated. Additionally, the western blot suggested that ligand of the immune checkpoint molecule PD-1, PD-L1, had significantly decreased expression following SAT2 overexpression within BxPC-3 and PANC-1 cells (Figure 7B). Meanwhile, as seen in the in vitro cell experimental results, SAT2 up-regulation enhanced the susceptibility of the PC cells to the cytotoxicity of human peripheral blood mononuclear cells while reducing immunosuppressive factor production, such as IL-10 and TGF-β1 (Figure 8A,B). Consequently, our proposition is that increased SAT2 expression within BxPC-3 and PANC-1 cells inhibits MAPK and PI3K/Akt pathway activation and reduces immunosuppression. Consequently, this inhibition results in diminished PC cell growth, invasion, and migration.

### 3.8. Overexpression of SAT2 Inhibits the Growth of PC Xenografts in Nude Mice

**Figure 9:**
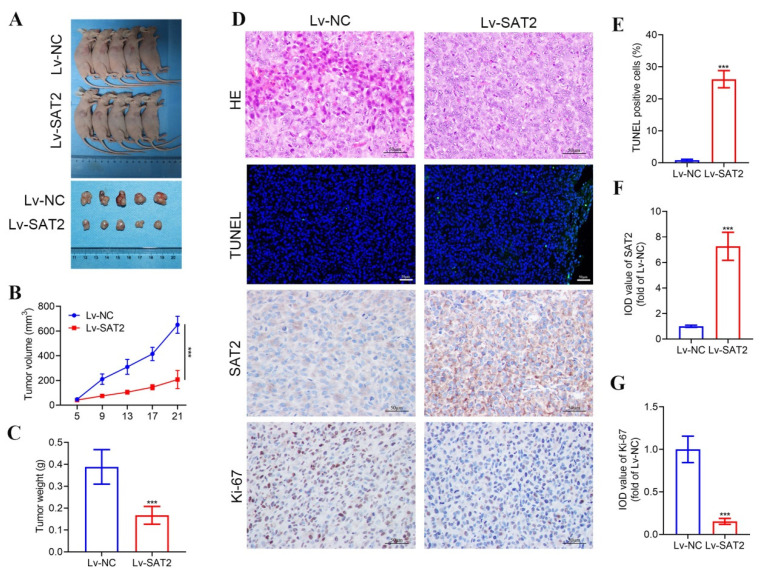
SAT2 high expression inhibited the growth of PC xenograft tumors in nude mice. (**A**) Diagram of tumor in nude mice; (**B**) tumor growth curve; (**C**) tumor weight; (**D**) results of HE, TUNEL, and IHC staining of the tumor tissue; bar = 50 μm; (**E**) percentage of apoptotic cells in tumor tissues; (**F**) SAT2 staining intensity in tumor tissues; (**G**) Ki-67 staining intensity in tumor tissues. *** *p* < 0.001 vs. Lv-NC group.

To investigate the role of SAT2 in PC progression, we conducted a xenograft tumor experiment in nude mice, followed by histopathological analyses including HE staining, TUNEL, and IHC assays on the excised tumor tissues. As shown in Figure 9A–C, SAT2 overexpression significantly suppressed tumor growth in nude mice. Furthermore, the pathological staining results indicated that in the Lv-NC group, the xenograft tumor tissue exhibited densely packed cells with marked cellular atypia. Tumor cell proliferation was observed in a nest-like or cluster-like pattern, with relatively few apoptotic cells. The expression level of the Ki-67 protein was elevated, whereas the expression of the SAT2 protein was comparatively low. In contrast to the Lv-NC group, the Lv-SAT2 group demonstrated an increased number of tumor cells with nuclear condensation, characterized by vacuolation or fragmentation. These cells were loosely arranged and possessed smaller nuclei, along with a higher number of apoptotic cells. Additionally, the protein expression levels of Ki-67 and PD-L1 were reduced, while SAT2 protein expression was significantly upregulated (Figure 9D–G, Supplementary Figure S1A). Collectively, these findings suggest that SAT2 overexpression suppresses the growth of xenograft PC tumors in nude mice.

## 4. Discussion

PC remains a formidable challenge due to its aggressive nature and limited therapeutic options. Our study identifies SAT2 as a potential tumor suppressor in PC, with its under-expression associated with larger tumor sizes, poorer prognoses, and altered immune dynamics. These findings align with the emerging evidence from other cancers, such as renal cell carcinoma, where SAT2 downregulation promotes tumor growth and resistance to therapy [7,17]. For instance, in renal cancer, SAT2 has been shown to enhance cisplatin sensitivity under hypoxia by degrading HIF-1α [7], suggesting a conserved tumor-suppressive mechanism that warrants further cross-cancer comparisons.

The prognostic value of SAT2 in PC is particularly noteworthy. Low SAT2 expression correlates with reduced OS and PFS, independent of other clinical factors such as age, grade, and staging. This positions SAT2 as a promising biomarker for risk stratification and potentially integration into nomograms for more personalized survival predictions. Compared to the established markers in PC, such as CA19-9 [18], SAT2’s high diagnostic AUC (0.882) from the TCGA data indicates a superior specificity, though validation in larger, prospective cohorts is needed to confirm its clinical utility over existing tools [19].

Functionally, our in vitro and in vivo experiments demonstrate that SAT2 overexpression inhibits PC cell proliferation, migration, and invasion while suppressing xenograft tumor growth and promoting apoptosis. These effects extend prior reports of SAT2’s antiproliferative role in other malignancies [7] and imply a therapeutic potential through SAT2 activation strategies, such as gene therapy or small-molecule agonists. However, unlike SAT1’s direct involvement in polyamine catabolism [6], SAT2’s weaker acetylation activity suggests alternative mechanisms, possibly through ubiquitin ligase complexes that target oncogenic proteins such as HIF-1α [7,8].

A key insight from our work is SAT2’s modulation of the PI3K/Akt and MAPK pathways, which are central to PC oncogenesis and immunotherapy resistance [20,21,22,23]. The overexpression of SAT2 reduced the phosphorylation of Akt, p38MAPK, and ERK, alongside the downregulation of downstream effectors such as Cyclin D1 and Vimentin, and the upregulation of the pro-apoptotic Bad and E-cadherin. Mechanistically, SAT2 may influence these pathways indirectly via its role in the ubiquitin-mediated degradation of regulatory proteins, such as HIF-1α, which can activate PI3K/Akt signaling under hypoxic conditions [8,17]. This degradation could disrupt upstream activators, leading to an attenuated pathway activity and the subsequent inhibition of cell survival and motility. Future studies should employ co-immunoprecipitation or proteomics to identify direct SAT2 interactors within these cascades, clarifying whether SAT2 acts as a scaffold or a modulator in PC-specific contexts.

Our findings also highlight SAT2’s impact on the tumor immune microenvironment (TIME), where low SAT2 expression correlates with increased M0 macrophages and decreased anti-tumor cells such as CD8^+^ T cells and plasma cells. This immunosuppressive profile aligns with PC’s characteristic evasion of host immunity [24,25,26] and contrasts with studies showing an enhanced survival linked to higher CD8^+^ T cell infiltration [27,28]. The negative correlation between SAT2 and PD-L1 (CD274) expression further suggests SAT2’s role in countering immune checkpoint-mediated suppression. Given that STAT3—a downstream target of PI3K/Akt—drives PD-L1 transcription [29], SAT2’s inhibition of Akt phosphorylation may indirectly suppress STAT3 activation, thereby reducing PD-L1 levels. Our research also confirmed that the overexpression of SAT2 reduces the levels of p-STAT3 and inhibits STAT3 activation in PC cells (Supplementary Figure S1B), thereby supporting the aforementioned hypothesis. Therefore, SAT2 may serve as a modulator of immunotherapy responsiveness and could potentially enhance the efficacy of anti-PD-L1 drug therapy in patients with high SAT2 expression.

In summary, SAT2 emerges as a multifaceted regulator in PC, suppressing tumor progression via PI3K/Akt-MAPK inhibition and fostering a less immunosuppressive TIME. These insights offer new avenues for targeted therapies, though the limitations include reliance on bioinformatics and cell lines, as well as the requirement for siRNA-mediated knockdown experiments targeting SAT2 to conclusively establish the specificity of the proposed mechanism. Future work, including in vivo mechanistic studies and clinical correlations, is therefore warranted. Elucidating SAT2’s precise interactions could unlock novel strategies to combat PC’s dismal prognosis.

## Figures and Tables

**Table 1:** The samples from TCGA, GEO and GTEx.

Name	Detail	Tumor (TCGA)	Normal (Source)
ACC	Adrenocortical carcinoma	77	77 (Adrenal gland , GTEx)
BLCA	Bladder urothelial carcinoma	406	19 (TCGA)
BRCA	Breast invasive carcinoma	1085	99 (TCGA)
CESC	Cervical squamous cell carcinoma and endocervical adenocarcinoma	306	13 (Cervix uteri, GTEx)
CHOL	Cholangio carcinoma	36	9 (TCGA)
COAD	Colon adenocarcinoma	448	41 (TCGA)
DLBC	Lymphoid neoplasm diffuse large B-cell lymphoma	47	47 (Whole blood, GTEx)
ESCA	Esophageal carcinoma	182	13 (TCGA)
GBM	Glioblastoma multiforme	167	163 (Brain cortex, GTEx)
HNSC	Head and neck squamous cell carcinoma	519	44 (TCGA)
KICH	Kidney chromophobe	66	25 (TCGA)
KIRC	Kidney renal clear cell carcinoma	531	25 (TCGA)
KIRP	Kidney renal papillary cell carcinoma	286	32 (TCGA)
LAML	Acute myeloid Leukemia	173	173 (Whole blood, GTEx)
LGG	Brain lower grade glioma	524	255 (Brain cortex, GTEx)
LIHC	Liver hepatocellular carcinoma	369	50 (TCGA)
LUAD	Lung adenocarcinoma	513	59 (TCGA)
LUSC	Lung squamous cell carcinoma	486	50 (TCGA)
MESO	Mesothelioma	87	87 (Heart atrial appendage, GTEx)
OV	Ovarian serous cystadenocarcinoma	426	180 (Ovary, GTEx)
PAAD	Pancreatic adenocarcinoma	178	4 (TCGA)
PCPG	Pheochromocytoma and paraganglioma	183	182 (Adrenal gland, GTEx)
PRAD	Prostate adenocarcinoma	499	52 (TCGA)
READ	Rectum adenocarcinoma	158	10 (TCGA)
SARC	Sarcoma	262	262 (Adipose subcutaneous, GTEx)
SKCM	Skin cutaneous melanoma	461	461 (Skin sun exposed lower, GTEx)
STAD	Stomach adenocarcinoma	408	36 (TCGA)
TGCT	Testicular germ cell tumors	139	137 (Testis, GTEx)
THCA	Thyroid carcinoma	512	59 (TCGA)
THYM	Thymoma	118	118 (Whole blood, GTEx)
UCEC	Uterine corpus endometrial carcinoma	544	35 (TCGA)
UCS	Uterine carcinosarcoma	57	57 (Uterus, GTEx)
UVM	Uveal melanoma	80	79 (EyeGEx retina, GTEx)
GSE15471		69 (GEO)	61 (GEO)
GSE16515		36 (GEO)	16 (GEO)
GSE62165		118 (GEO)	13 (GEO)

**Table 2:** Differential expression of SAT2 gene and other clinicopathological parameters in PAAD.

	PAAD			
Variable	Cases	High	Low	* p * Value
Age (years)				0.2334
<60	55 (31.07%)	15 (21.43%)	40 (72.73%)	
≥60	122 (68.93%)	30 (24.59%)	92 (75.41%)	
Gender				0.8908
Female	80 (45.20%)	22 (27.50%)	58 (72.50%)	
Male	97 (54.80%)	23 (23.71%)	74 (76.29%)	
TNM stage				0.4029
I–II	167 (94.35%)	42 (25.15%)	125 (74.85%)	
III–IV	8 (5.65%)	2 (25.00%)	6 (75.00%)	
T stage				0.0036
T1–T2	31 (17.61%)	13 (41.94%)	18 (58.06%)	
T3–T4	145 (82.39%)	31 (21.38%)	114 (78.62%)	
N stage				0.3670
N0	49 (65.80%)	12 (62.61%)	37 (37.39%)	
N1	123 (34.20%)	32 (60.82%)	91 (39.18%)	
M stage				0.2503
M0	79 (45.66%)	15 (18.99%)	64 (81.01%)	
MX	94 (54.34%)	29 (16.76%)	65 (83.24%)	
Grade				0.386
G1–G2	126 (71.19%)	36 (28.57%)	90 (71.43%)	
G3–G4	51 (28.81%)	8 (15.69%)	43 (84.31%)	
New tumor				0.0674
No	86 (55.13%)	25 (26.07%)	61 (73.93%)	
Yes	70 (44.87%)	13 (18.57%)	47 (81.43%)	
Smoking history				0.6803
≤15 years	17 (29.82%)	3 (17.65%)	14 (82.35%)	
>15 years	40 (70.18%)	9 (22.50%)	31 (77.50%)	
Alcohol history				0.5174
Yes	102 (61.45%)	27 (26.47%)	75 (73.53%)	
No	64 (38.55%)	16 (25.00%)	48 (75.00%)	

N, nodal metastasis status; T, tumor size; M, tumor metastasis; new tumor, new tumor events after initial treatment.

## Data Availability

The original contributions presented in this study are included in the article/Supplementary Materials. Further inquiries can be directed to the corresponding author(s).

## Supplementary Materials

The supporting information can be downloaded at: https://www.mdpi.com/article/10.3390/cimb47100872/s1.
