# Supplementary material for: The Tumor-Suppressive Role of SAT2 in Pancreatic Cancer: Involvement in PI3K/Akt-MAPK Pathways and Immune Modulation
Source: Curr Issues Mol Biol. 2025 Oct 21;47(10):872. doi: 10.3390/cimb47100872 (PMC12562838; doi:10.3390/cimb47100872)
Supplement: Supplementary file 1 [file cimb-47-00872-s001.zip › cimb-3835398-Supplementary materials - UPDATE.pdf]

The Tumor-Suppressive Role of SAT2 in Pancreatic Cancer: Involvement in PI3K/Akt-MAPK Pathways and Immune Modulation

Ben Zhao [1], Lu Wang [1], Rui Fang [2,3], Xiaoxiao Luo [1] and Lu Zhang [1,*]

1. Department of Oncology, Tongji Hospital, Tongji Medical College, Huazhong University of Science and Technology, Wuhan 430030, China

2. Bridge Institute of Experimental Tumor Therapy, West German Cancer Center, University Hospital Essen, University of Duisburg-Essen, 45147 Essen, Germany

3. Division of Solid Tumour Translational Oncology, German Cancer Consortium (DKTK), Partner Site Essen, a Partnership Between German Cancer Research Centre (DKFZ) and University Hospital Essen, 45147 Essen, Germany

Conflicts of Interest: The authors report no conflict of interest.

*** Correspondence author:**

Mr. Lu Zhang, MD, Department of Oncology, Tongji Hospital, Tongji Medical College, Huazhong University of Science and Technology, No.1095 Jiefang Avenue, Qiaokou District, Wuhan 430000, Hubei Province, the People's Republic of China. E-mail: zhanglu@tjh.tjmu.edu.cn.

Figure legends

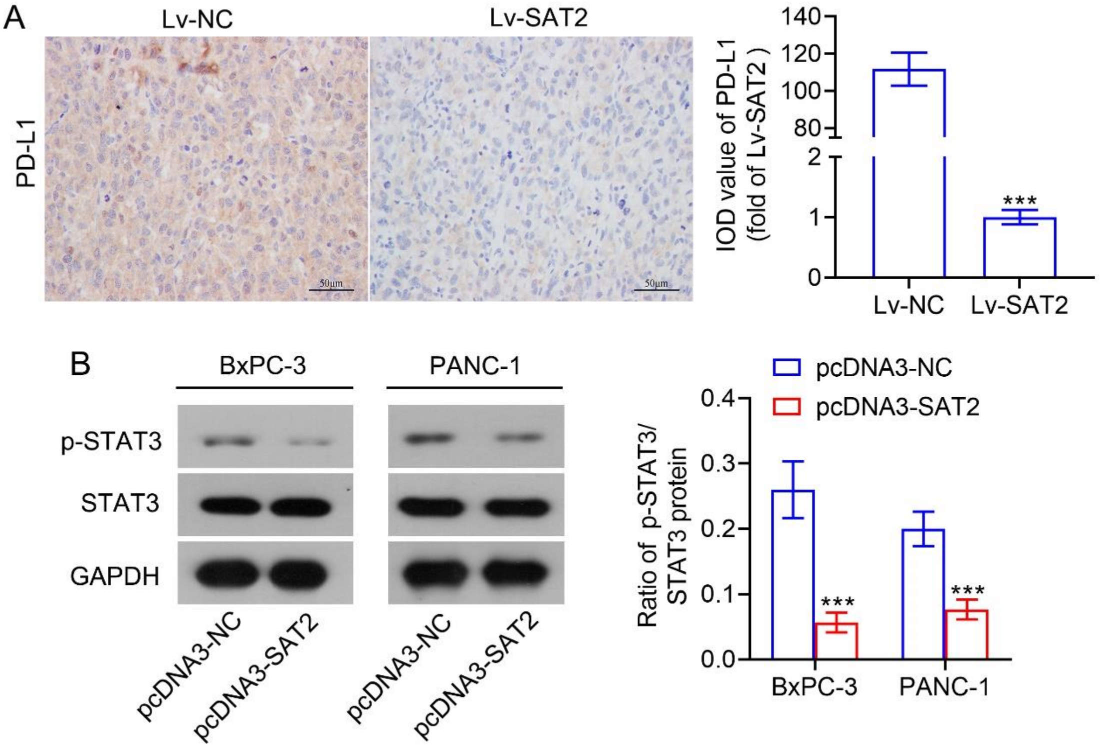

Supplementary Figure S1. The effects of SAT2 overexpression on PD-L1 expression in xenograft tumors of nude mice and on STAT3 pathway activation in PC cells

(A) The expression of PD-L1 in the xenograft tumor tissues of nude mice was detected by immunohistochemical assay. ***p<0.001 compared with the Lv-NC group.（B）The expression levels of p-STAT3 and STAT3 proteins in transfected PANC-1 and BxPC-3. ***p<0.001 compared with the pcDNA3-NC group.
